# Use of Bulk Segregant Analysis for Determining the Genetic Basis of Azole Resistance in the Opportunistic Pathogen *Aspergillus fumigatus*

**DOI:** 10.3389/fcimb.2022.841138

**Published:** 2022-04-05

**Authors:** George D. Ashton, Fei Sang, Martin Blythe, Daniel Zadik, Nadine Holmes, Sunir Malla, Simone M. T. Camps, Victoria Wright, Willem J. G. Melchers, Paul E. Verweij, Paul S. Dyer

**Affiliations:** ^1^ School of Life Sciences, University of Nottingham, Nottingham, United Kingdom; ^2^ DeepSeq, Centre for Genetics and Genomics, Queen’s Medical Centre, University of Nottingham, Nottingham, United Kingdom; ^3^ Department of Medical Microbiology, Radboud University Medical Centre, Nijmegen, Netherlands

**Keywords:** antifungal, itraconazole, genomics, filamentous fungi, next-generation sequencing, CYP51

## Abstract

A sexual cycle was described in 2009 for the opportunistic fungal pathogen *Aspergillus fumigatus*, opening up for the first time the possibility of using techniques reliant on sexual crossing for genetic analysis. The present study was undertaken to evaluate whether the technique ‘bulk segregant analysis’ (BSA), which involves detection of differences between pools of progeny varying in a particular trait, could be applied in conjunction with next-generation sequencing to investigate the underlying basis of monogenic traits in *A. fumigatus*. Resistance to the azole antifungal itraconazole was chosen as a model, with a dedicated bioinformatic pipeline developed to allow identification of SNPs that differed between the resistant progeny pool and resistant parent compared to the sensitive progeny pool and parent. A clinical isolate exhibiting monogenic resistance to itraconazole of unknown basis was crossed to a sensitive parent and F1 progeny used in BSA. In addition, the use of backcrossing and increasing the number in progeny pools was evaluated as ways to enhance the efficiency of BSA. Use of F1 pools of 40 progeny led to the identification of 123 candidate genes with SNPs distributed over several contigs when aligned to an A1163 reference genome. Successive rounds of backcrossing enhanced the ability to identify specific genes and a genomic region, with BSA of progeny (using 40 per pool) from a third backcross identifying 46 genes with SNPs, and BSA of progeny from a sixth backcross identifying 20 genes with SNPs in a single 292 kb region of the genome. The use of an increased number of 80 progeny per pool also increased the resolution of BSA, with 29 genes demonstrating SNPs between the different sensitive and resistant groupings detected using progeny from just the second backcross with the majority of variants located on the same 292 kb region. Further bioinformatic analysis of the 292 kb region identified the presence of a *cyp51A* gene variant resulting in a methionine to lysine (M220K) change in the CYP51A protein, which was concluded to be the causal basis of the observed resistance to itraconazole. The future use of BSA in genetic analysis of *A. fumigatus* is discussed.

## Introduction

*Aspergillus fumigatus* is a ubiquitous fungus in the environment where it is primarily saprotrophic in nature, breaking down organic matter found in soil and compost. However, as a result of the inhalation and growth of airborne conidia produced by the fungus, *A. fumigatus* can also act as an opportunistic pathogen in immunocompromised individuals where infection can lead to mortality rates as high as 90% (Dagenais and Keller, 2009; Brown et al., 2012). Co-infection by *A. fumigatus* has also been reported recently as a complicating factor in COVID19 cases (e.g. Arastehfar et al., 2020; Van Arkel et al., 2020). The fungus was long considered a purely asexual organism but genome analysis led to the discovery of a suite of genes correlated with sexual reproduction, which suggested the potential for sexual reproduction (Galagan et al., 2005; Nierman et al., 2005; Paoletti et al., 2005). A functional sexual cycle was subsequently revealed and it was recently shown that sexual fertility was present in the vast majority of global isolates examined (O’Gorman et al., 2009; Swilaiman et al., 2020).

Treatment of *A. fumigatus* has proven challenging due to the common evolutionary origins of the fungal and animal kingdoms, meaning that there are difficulties finding therapeutics that target the fungal infection whilst minimising side effects experienced by humans. Consequently, only a limited number of drug classes are currently available to treat invasive aspergillosis. These include the polyenes (targeting membrane integrity), the echinocandins (targeting chitin synthesis), and the azoles (targeting membrane biosynthesis). All these therapeutics have fungal-specific cellular targets (Sanglard and Odds, 2002; Cowen et al., 2015). The current frontline treatment for invasive aspergillosis involves the use of azole antifungals, due to the detrimental side effects often associated with polyene treatment, such as acute renal failure from amphotericin B (Patterson et al., 2016). The azole class of drugs acts on *A. fumigatus* by binding to, and inhibiting the functioning of the CYP51A protein which is essential for ergosterol synthesis, with ergosterol being a key component of fungal cell membranes. Therefore, azole treatment interferes with membrane formation by repressing the ergosterol biosynthesis pathway, which subsequently results in inhibition of fungal growth and eventual cell death with carbohydrate patch formation in the cell wall (Kelly et al., 1995; Alcazar-Fuoli et al., 2008; Cowen et al., 2015).

Azoles have been used since the 1980s for medical treatment of fungal infections. However, there has been a rising trend over the last few decades of azole-resistant isolates of *A. fumigatus* being identified from both clinical and environmental settings (Verweij et al., 2020). Since the first report of azole resistance, this has now become a worldwide phenomenon with associated risk (Chowdhary et al., 2014). Azole resistance is thought to have evolved due to selection both in clinical settings and also in the broader environment, where widespread use of azoles for agricultural and preservative applications may drive selection for resistance. The managed environment therefore represents a potential reservoir of azole-resistant isolates which may infect humans (Verweij et al., 2020). Indeed, there is very recent evidence from population genomic studies for such infection of humans by drug resistant *A. fumigatus* from the environment (Rhodes et al., 2022).

Studies have been made into the genetic basis of azole resistance in *A. fumigatus.* The majority of resistance has been found to be due to mutations in the target *cyp51A* gene coding region and/or its associated promoter region. Mutations in the coding region can lead to structural changes in the CYP51A protein, resulting in reduced binding efficiency of azoles (e.g. DiazFGuerra et al., 2003; Howard et al., 2009; Camps et al., 2012). Meanwhile, mutation in the promoter region, typically *via* tandem repeat insertions, can lead to gene overexpression (often in combination with point mutations in the *cyp51A* coding region) (e.g. Mellado et al., 2007; Camps et al., 2012; Snelders et al., 2015; Hare et al., 2017). Although mutations in *cyp51A* are attributed as a primary cause of resistance in *A. fumigatus* to azoles, there have been several reports of resistant isolates which did not contain a mutation in the *cyp51A* gene. For example, a survey of clinical isolates from Manchester (UK) showed that approximately 50% of resistant isolates had no mutation in c*yp51A* or its promoter (Fraczek et al., 2013). Indeed, more recently there have been reports of other mutations that can lead to a resistance phenotype. These include mutations in the *hapE* gene which can result in increased expression of *cyp51A* (Gsaller et al., 2016), mutations in the *hmg1* gene which can also lead to increased internal ergosterol levels (Losada et al., 2015; Rybak et al., 2019), and altered expression of efflux transporters (Fraczek et al., 2013; Paul et al., 2013; Paul et al., 2017). However, there remain a large number of reports of isolates where the mechanism of resistance is unknown (e.g. Arendrup et al., 2010; Denning et al., 2011; Lockhart et al., 2011; Ozmerdiven et al., 2015; Abdolrasouli et al., 2018; Sharma et al., 2019). It remains vital to identify the genetic basis of such mutations in order to facilitate the design of diagnostics to improve treatment outcome in those suffering from invasive aspergillosis.

Genetic analysis exploiting the sexual cycle as a tool has only recently become possible in *A. fumigatus* following the identification of a heterothallic (obligate outbreeding) sexual cycle by O’Gorman et al. (2009), with isolates classified as of either mating type *MAT1-1* or *MAT1-2.* This has meant that several experimental opportunities are now possible. First, it means that where a particular phenotypic trait, such as azole resistance, has been identified in an isolate of *A. fumigatus*, it is possible to determine the Mendelian basis of this phenotype by setting up sexual crosses with a suitable partner (lacking the phenotype) and analysing the progeny for the phenotype. Thus, it is possible to determine if the trait observed is monogenic or polygenic in nature from the segregation patterns in the sexual progeny (Ashton and Dyer, 2016). For example, in the case of antifungal resistance, the progeny would be predicted to exhibit a near 1:1 ratio of drug resistant to sensitive phenotype in the scenario of a monogenic resistance, whereas for resistance with a polygenic basis a continuous spectrum of phenotypes would be predicted to be present in the sexual progeny (Dyer et al., 2000).

Second, the discovery of a sexual cycle in *A. fumigatus* has meant that a range of other methods reliant on the analysis of sexual progeny have now become possible. Of particular significance is that where an unknown trait is determined to be monogenic in origin, the method bulked (later abbreviated to ‘bulk’) segregant analysis (BSA) can be employed to identify the region of the genome in which a novel mutation(s) is contained and thereby identify candidate genes of interest (Michelmore et al., 1991). The process involves sexually crossing a parent with the phenotype of interest (e.g. azole resistance) with an isolate (of the opposite mating type) lacking the phenotype. The progeny are harvested and separated into two groups based on the presence or absence of the trait of interest. These groups are then compared as two discrete bulked genomic samples (or pools). In the classical application of BSA, numerous DNA fingerprint markers are then generated in order to detect a marker linked to the trait of interest (Michelmore et al., 1991; Zhu et al., 1996; Jurgenson et al., 2002; Baird et al., 2008; Dettman et al., 2010). More recently, in the field of fungal genetics BSA has been updated to incorporate next-generation sequencing (NGS) and has been employed with pools not necessarily from classical sexual crosses. Such studies have allowed accurate identification of regions and/or single nucleotide polymorphisms (SNPs) of difference between pools exhibiting or lacking the trait of interest (Wenger et al., 2010; Pomraning et al., 2011; Nowrousian et al., 2012; Niu et al., 2016; Heller et al., 2016; Heller et al., 2018; Goncalves et al., 2019). From this, further experimental work can be performed as necessary to pinpoint and characterize a gene and/or SNP of interest.

The present study was therefore undertaken to investigate the possibility of utilising BSA in combination with NGS in *A. fumigatus* as a tool for determining the genetic basis of traits of interest where the trait is known to be monogenic in basis but the causal gene/SNP is unknown. As a model we used sexual crossing to study the genetic basis of resistance to azole antifungal drugs found in a clinical isolate of *A. fumigatus* with no supposed known resistance genotype. The work included evaluation of the use of different numbers of backcrosses, and increasing the number of progeny used in the progeny pools, to enhance and optimise the efficiency of BSA in *A. fumigatus.* This was in order to reduce the presence of noise in the genetic data produced, which has previously been reported as a difficulty associated with BSA (Pomraning et al., 2011).

## Materials and Methods

### Strains, Culture Conditions and Maintenance

The parental *Aspergillus fumigatus* isolates used were: 47-308 (synonym v68-66; *MAT1-2*) that had been shown to exhibit monogenic resistance to itraconazole in previous crossing work, which was both stable and of a relatively high level, and did not exhibit any known mutation in the *cyp51A* gene (O’Gorman and Dyer, unpublished results; S. Camps, personal communication); and 47-51 (synonym AfIR974; *MAT1-1*), a highly fertile azole sensitive isolate from air sampling in Dublin, Ireland (O’Gorman et al., 2009). Both 47-308 and 47-51 were stored under liquid nitrogen as 20% glycerol stocks (1:5 in sterile water) in the University of Nottingham BDUN collection. When required, isolates were cultured on Aspergillus complete medium (Paoletti et al., 2005) and grown for 7 days at 28 °C before use.

### Sexual Cycle and Progeny *MAT* Analysis

For comprehensive details of methods used for sexual crossing, ascospore isolation and mating-type analysis of progeny please see the protocols paper of Ashton and Dyer (2019). In brief, sexual crosses were made between complimentary *MAT1-1* and *MAT1-2* mating partners using a barrage method on oatmeal agar as described by O’Gorman et al. (2009). After three months ascospores were harvested from cleistothecia and the mating-type of progeny was determined using a multiplex PCR *MAT* assay (Paoletti et al., 2005). Serial backcrosses were then set up between *MAT1-2* progeny that were azole resistant with the original 47-51 (*MAT1-1*) azole sensitive parental isolate. To ensure crossing success, matings with at least four different *MAT1-2* azole resistant progeny were set up with the 47-51 parent at each backcross (although only offspring from one successful cross was required).

### Itraconazole Resistance Screening

Azole sensitivity or resistance of parental *A. fumigatus* isolates and subsequent progeny were determined using the ETEST^®^ method in accordance with manufacturer’s instructions (Biomerieux). Spore suspensions of each isolate to be tested were made and adjusted to a concentration of 1x10^6^ spores ml^-1^ in 0.05% Tween 80 (Sigma-Aldrich) and spread using a cotton swab onto RPMI 1640 (Sigma-Aldrich) supplemented with 2% glucose (Fischer Scientific) and adjusted to a final pH of 7.0 with morpholinepropanesulphonic acid (MOPS) buffer (Sigma-Aldrich). Once dried, an itraconazole ETEST strip was placed at the center of the Petri dish and incubated at 35 °C for 48 h. The minimum inhibitory concentration (MIC) was determined as the maximum point on the ETEST strip that the fungal culture grew to.

### DNA Extraction and Preparation of Bulk Segregant Pools

To prepare for each round of bulk segregant analysis, progeny were separated into two discrete groups (pools): the first showing the resistance profile of interest (as observed in parental isolate 47-308) and the second showing itraconazole sensitivity (as observed in parental isolate 47-51). Separate rounds of BSA were undertaken with progeny from the initial sexual cross (i.e. F1 progeny from 47-51 x 47-308) and the 3rd backcross and the 6th backcross (BC3 and BC6, respectively), with 40 resistant progeny and 40 sensitive progeny being isolated and used for subsequent DNA pool analysis. DNA was extracted using a Qiagen Plant DNA extraction kit following the manufacturer’s instructions for adaptation for filamentous fungi (Qiagen). DNA quality and quantity for genome sequencing was then assessed by gel electrophoresis, Nanodrop and Qubit methodologies (Thermo Fisher), respectively In addition, BSA was later undertaken with progeny from the 2nd backcross (BC2), but this time 80 resistant and 80 sensitive progeny were isolated and used for subsequent DNA pool analysis. For each round of BSA, DNA was extracted from progeny and DNA pooled together based on resistance or sensitivity phenotype. For each DNA pool, 2.5 µl (for F1, BC3 and BC6) or 1.25 µl (for BC2) of each appropriate progeny DNA sample was added together to form a final pool of 100 µl at a concentration of 50 ng µl^-1^.

### Genome Sequencing and Bulk Segregant Approach

Whole genome sequencing was performed by DeepSeq at the Queen’s Medical Centre (University of Nottingham, UK). Libraries were prepared from 100 ng to 1 ug of input DNA using the NEBNext Ultra kit (NEB, UK) following the manufacturer’s standard protocol. Libraries were then pooled and sequenced using the MiSeq instrument with the MiSeq Reagent Kit v3 (600 cycles) (Illumina, USA) to generate 2 x 300bp paired end reads, with three replicates used per parent or progeny pool and a target of ca. 120-fold sequencing depth. As indicated above, different rounds of BSA were undertaken after one round of sexual crossing (F1) and then after the 2nd, 3rd and 6th round of backcrossing (BC2, BC3, BC6, respectively). Rules were applied to the arising sequence data following the principles of BSA i.e. that any marker linked to the phenotype of interest should only be present in the resistant pool and resistant parent, and be absent from the sensitive pool and the sensitive parent. Any marker should also be absent from the (sensitive) wild-type reference genomes (**Supplementary Figure 1**).

For the first two rounds of BSA, using progeny from the F1 and the 3rd backcross, reference genomes of *A. fumigatus* available from the NCBI (isolate AF293; Genbank accession NC_007194.1) and JGI (isolate A1163; Genbank accession ABDB00000000) databases were used to align sequence reads. However, due to only minor differences observed between sequence data from both reference databases, for the subsequent BSA of progeny from the 2nd backcross and 6th backcross, only the JGI A1163 reference database was used for alignment (https://mycocosm.jgi.doe.gov/Aspfu_A1163_1/Aspfu_A1163_1.home.html).

### Bioinformatic Methods and Candidate Gene Identification

The sequencing analysis pipeline aimed to identify any SNPs evident between the pools and parents, and then data was narrowed down to focus specifically on SNPs present in coding regions that might impact on protein function. In summary, the BSA involved five steps. First, reads were subject to quality control and trimming. Second, variant information for each replicate was identified by aligning the reads to each of the JGI and NCBI versions of the *A. fumigatus* genomes independently, and subsequently calling SNP variants from the reference genomes. Third, the variant information for each group of three sample replicates was compared, and the homozygous variants common between the replicates were determined. This was achieved by processing the variant VCF files using the program BedTools (Quinlan, 2014). Next, the common variants (i.e. exhibiting SNPs from the reference genomes) from each group were then compared to those of every other group, so that the variants present in both the resistant strain progeny and parent samples, but absent in both the susceptible strain progeny and parent samples, were identified. This was achieved using either VCFtools (Danecek et al., 2011) or BCFtools (Li, 2011). Finally, the predicted effect of the selected variants on the coding regions of the corresponding genome sequence was determined against the available genome annotation information using the program SnpEff (Cingolani et al., 2012). A check was also made whether the causative mutation might be due to deletion of a region of the genome. Due to the time interval between different sets of sample processing, slightly different methods were used with the pipeline for the analysis being refined as described below. Each step is now described in more detail as follows.

#### Raw Read Analysis and Trimming

Reads were initially assessed for quality using the FastQC read analysis tool (http://www.bioinformatics.babraham.ac.uk/projects/fastqc/). Raw reads were then either trimmed for adapters using Scythe (https://github.com/vsbuffalo/scythe) and then trimmed again for quality using Sickle (https://github.com/najoshi/sickle; Joshi and Fass, 2011) using default settings.

#### Read Mapping

Reads were aligned to the genome sequences of *A. fumigatus* sourced from the JGI (Aspfu_A1163_1) and separately to the Af293 genome sequence from Genbank (NCBI BioProject: PRJNA14003) using BWA-MEM (http://bio-bwa.sourceforge.net; Li and Durbin, 2009; Li and Durbin, 2010) with default alignment parameters. The genome sequences were masked for repeat elements in fungi using RepeatMasker (http://www.repeatmasker.org; Smit and Hubley, 2008-2015). Resultant SAM files were then sorted and converted to BAM files using SAMtools (http://samtools.sourceforge.net; Li et al., 2009). SAMtools was also used to filter for correctly and uniquely mapped reads. Read duplicates were identified and marked within the BAM file using Picard Tools (http://broadinstitute.github.io/picard).

#### Variant Calling

SNPs and InDels (insertions & deletions) were called using the GATK (Genome Analysis Tool Kit) tool GATK HaplotypeCaller (https://gatk.broadinstitute.org/hc/en-us; McKenna et al., 2010). Initial variant calls were then filtered using the GATK VariantFiltration program. Variant calls were recorded in VCF file format, whilst filtering and removing variants corresponding to low coverage regions and low quality read alignments. For later BC2 and BC6 analyses the VCF files were compressed with bgzip to save storage space. Common variants shared among all three replicates for each individual group (i.e. resistant or sensitive parent, and resistant or sensitive pools) were determined using BCFtools (http://github.com/samtools/bcftools; Li, 2011). Common variants for each group were then used in the downstream analysis, including the key identification of variants that were present in the 47-308 parent and progeny resistant pool but not in the 47-51 parent and progeny sensitive pool. This comparison of the variants in each set was conducted using the isec tool of BCFtools. Furthermore, it was considered that the identified and selected SNP variants, as defined above, could be filtered further by a comparison to variants found between the two genomes (JGI & NCBI) as both the Af293 and A1163 genome reference strains do not contain the resistant trait. Other comparisons between group were also calculated. Variant ratios for a region of interest were calculated according to read depth to allow investigation of the extent of homozygosity and heterozygosity at a given SNP, with values calculated as the ratio of the average coverage supporting that variant compared to the average total coverage at that given position across the three replicates in the progeny pools. For earlier F1 and BC3 analyses the raw VCF files were analysed directly (i.e. without compression) when seeking to identify differences between the 47-308 parent and progeny resistant pool versus the 47-51 parent and progeny sensitive pool, using the tool vcf-isec, part of the suite of VCFtools (https://vcftools.github.io; http://vcftools.sourceforge.net/perl_module.html#vcf-isec; Danecek et al., 2011).

#### Variant Effect Prediction

The filtered and selected SNP and InDel variants common to the resistant groups but absent in the sensitive groups (generated by GATK, with a designation of “PASS”), were then processed with SnpEff (3.3b; http://snpeff.sourceforge.net; Cingolani et al., 2012) in order to predict the functional effect of each variant with regard to gene coding regions. The effected genes were defined and reported with the output of SnpEff. The genes with variants that effected the protein product (frame shift, missense & non-synonymous) were plotted along the length of the gene.

#### Zero Coverage Regions

An examination of the regions of the genome with no coverage in mapped reads was conducted to assess whether the causative resistant mutation might be due to a deletion of a particular genome region. Using Bedtools, the BAM file for each replicate was processed and regions with no coverage in aligned reads using BedTools (https://bedtools.readthedocs.io; Quinlan, 2014). The genome bases with no coverage that were common to each set of replicates within a group were initially determined, and these group sets were subsequently compared. The results were recorded in BED format.

## Results

### ETEST and Segregation Pattern

**Figure 1:**
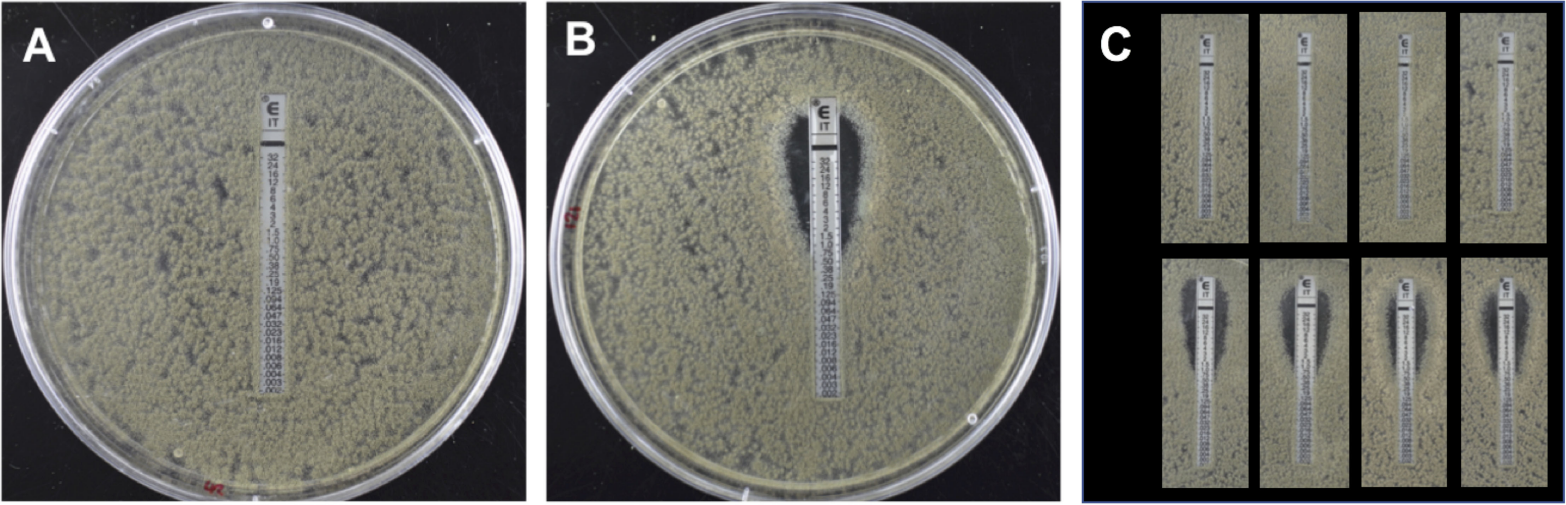
Itraconazole ETEST sensitivity testing of *Aspergillus fumigatus* strains 47-308 **(A)** and 47-51 **(B)** and progeny from back crossing. The ETEST has a concentration of 32 mg L^-1^ itraconazole at the top of the strip which decreases down the strip towards the bottom. **(A)** Isolate 47-308 shows full growth over the range of itraconzole concentrations, indicating a resistance MIC of >32 mg L^-1^. **(B)** Isolate 47-51 shows inhibition of growth at itraconazole concentrations above 0.75 mg L^-1^ indicating a sensitive phenotype. **(C)** Representative ETEST results of progeny isolated from the third backcross between a resistant progeny isolate and the itraconazole sensitive parental isolate 47-51. Results demonstrate a 1:1 segregation of the resistant (top row) and sensitive (bottom row) phenotype.

**Figure 2:**
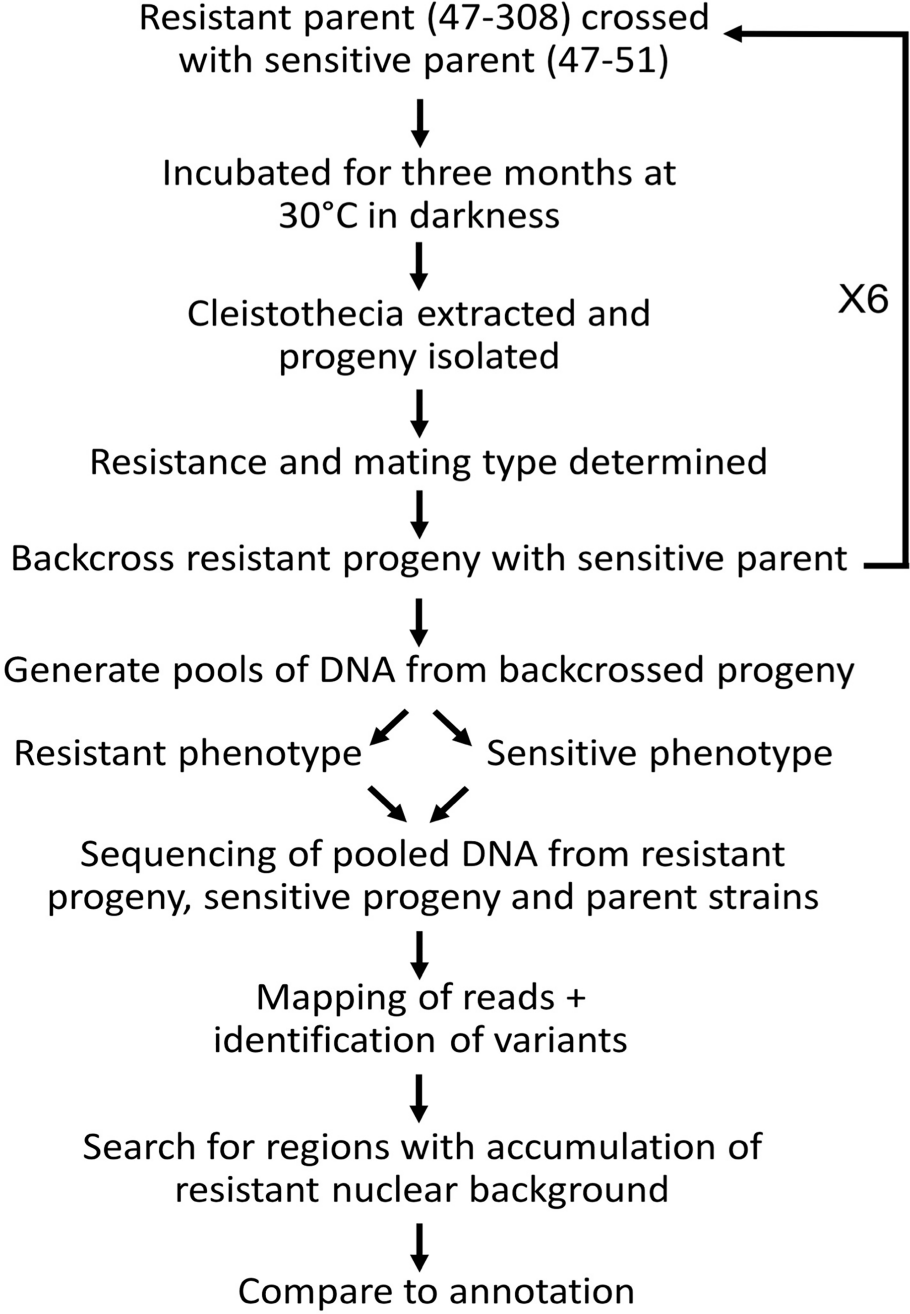
Methodology of bulk segregant analysis used in the present study. An azole resistant isolate (47-308) carrying an unknown mutation was sexually crossed with a sensitive isolate (47-51). F1 progeny were collected and up to six rounds of backcrossing with the sensitive parent undertaken with arising resistant progeny. At certain stages the collected progeny were separated into azole resistant and sensitive pools, which were genome sequenced in order to identify genomic regions within the resistant progeny containing a candidate gene(s) of interest.

The ETEST method provided a reliable method to screen parents and progeny for resistance or sensitivity to itraconazole. For each itraconazole resistant isolate, including parental isolate 47-308 and appropriate progeny, a minimum inhibitory concentration (MIC) of >32 mg L^-1^ was observed. By contrast, an MIC of ≤0.75 mg L^-1^ was evident in sensitive isolates, including parental isolate 47-51 and appropriate progeny. Furthermore, the segregation of the resistance phenotype remained at a near 1:1 ratio throughout each sexual cross, confirming the monogenic nature of this resistance phenotype (**Figure 1**). This allowed the subsequent bulk segregant analysis (BSA) to be carried out of F1 or back crossed (BC) progeny pools in combination with next-generation sequencing (NGS). All matings with different *MAT1-2* azole resistant progeny consistently produced apothecia with the sensitive 47-51 parent, so progeny from a representative single back cross were selected at each round for further backcrossing. BSA was undertaken first with 40 progeny per respective pool (F1, BC3, BC6) and then with 80 progeny (BC2). For the initial BSA involving pools from the F1 and BC3 progeny the bioinformatic analysis used both the NCBI and JGI reference genomes for *A. fumigatus* (A1163 and Af293; data listed as contigs and chromosomes, respectively) whereas later BC6 and BC2 BSA bioinformatic analyses used only the JGI A1163 reference database given that that only minor differences were observed between results drawing on sequence data from either reference database. Parental isolate 47-51 exhibited 48,882 SNP variants, whilst parental isolate 47-308 exhibited 9,554 variants, from the A1163 reference genome, respectively, with the two parents themselves differing by at least 39,814 SNPs based on alignment to the A1163 reference genome (**Supplementary Figure S2**). The work flow used in the bulk segregant analysis is summarised in **Figure 2** for clarity, using either 40 or 80 sexual progeny for the BSA pools. It was noted early on in the bioinformatic analysis that the zero-coverage regions defined in the S-pool group were the same as the R-pool group, and therefore no discriminatory deletion responsible for azole resistance was identified.

### Use of BSA and NGS With 40 Sexual Progeny per DNA Pool

#### Bulk Segregant Analysis of F1 Progeny

Bioinformatic analysis of genomic data from the 40 pooled resistant progeny in comparison to the sensitive parent, 40 pooled sensitive progeny and JGI reference genome A1163 revealed 123 genes demonstrating SNPs between the different sensitive and resistant groupings (**Table 1**). These differences, as well as SNPs in non-coding regions, were spread across 11 separate contigs in the genome with contig DS_499598 showing the largest number of differences (26% of the total) compared to the other contigs (**Supplementary Figure S3**). Meanwhile, comparison to the NCBI reference genome Af293 revealed 150 genes exhibiting SNPs between the different sensitive and resistant groupings. These differences were spread between 8 chromosomes with chromosome 4 having the largest number of differences (34% of the total). Details of the position, gene ID, reference and SNP variants for both JGI and NCBI reference genome comparisons are provided in Ashton (2018). Given the high number of candidate genes and the fact that no single genome region was identified from the BSA, successive rounds of backcrossing were then undertaken in an attempt to more precisely identify a single region of interest.

**Table 1:** Summary of *A. fumigatus* bulk segregant analysis (BSA) for identification of the genetic basis of azole resistance in isolate 47-308.

Stage that BSA undertaken	Number of progeny used in each BSA pool	Number of variant genes identified	Number of contigs with SNP variants	Number of SNP variants	Sequencing depth of progeny pools
F1	40	123	11	561	124.4-126.8
Backcross 3	40	46	6	493	83.9-96.7
Backcross 6	40	22	6	135	145.8-193.5
Backcross 2	80	29	7	170	103.1-211.2

BSA was undertaken with different combinations of progeny numbers and at different stages of crossing between sensitive and resistant parents or backcrossed progeny. Results refer to SNP differences between the azole resistant pool and parent when compared to the azole sensitive pool and parent using the A1163 JGI reference genome for bioinformatic analysis.

#### Bulk Segregant Analysis of Third Backcross and Sixth Backcross Progeny

**Figure 3:**
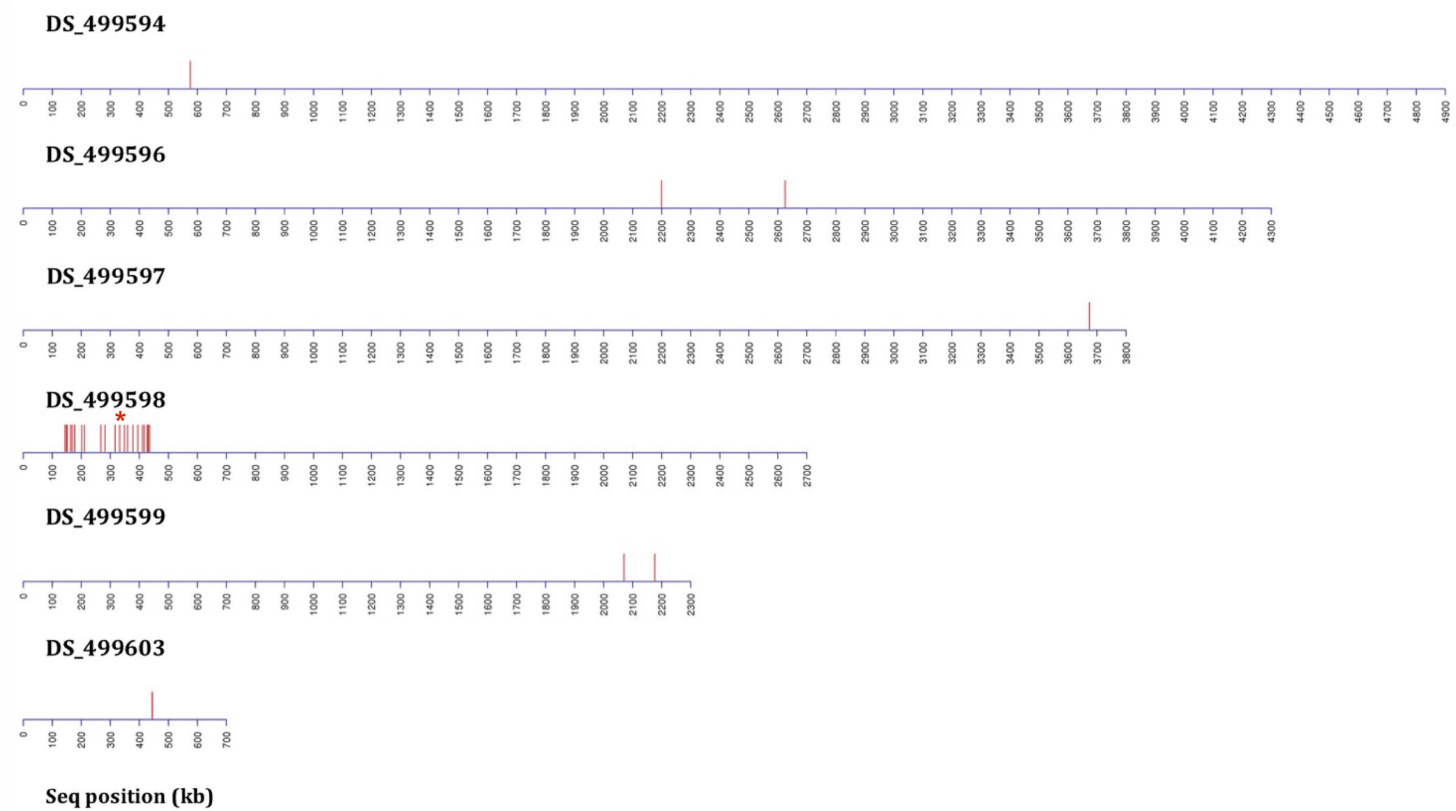
Distribution plots of SNP variants identified from bioinformatic analysis mapped onto *A. fumigatus* JGI reference A1163 genome contigs (not to scale) from bulk segregant analysis (BSA) applied following six rounds of backcrossing (BC6) using 40 progeny per BSA pool. Each red bar represents one or more (if in close proximity) variant sites exhibiting a consistent difference between the sensitive and resistant groupings. Data includes sites present in gene coding and non-coding regions. The red asterisk above contig DS_499598 indicates the position of the M220K causal variant in *cyp51A*.

Six rounds of backcrossing were performed in total, and BSA, in combination with NGS, undertaken using pooled sets of 40 sensitive and 40 resistant progeny derived from the 3^rd^ and 6^th^ backcrosses. Bioinformatic analysis of genomic data from the resistant progeny from the 3^rd^ backcross (BC3) in comparison to the sensitive parent, sensitive progeny and the reference A1163 genome, revealed 46 genes demonstrating SNPs between the different sensitive and resistant groupings (**Table 1**). The variants, as well as SNPs in non-coding regions, were spread over 6 contigs, with 89% of the variants located on contig DS_499598 (**Supplementary Figure S4**). Meanwhile, comparison to the NCBI reference Af293 revealed 60 genes exhibiting SNPs between the different sensitive and resistant groupings. These differences were spread over 8 chromosomes, with chromosome 4 showing 83% of differences in comparison to other contigs. The position, gene ID, reference and SNPs for both JGI and NCBI comparisons are described in detail in Ashton (2018). Finally, bioinformatic analysis of genomic data from of the resistant progeny from the 6^th^ backcross (BC6) in comparison to the sensitive parent, sensitive progeny and the reference A1163 genome revealed 22 genes demonstrating SNPs between the different sensitive and resistant groupings (**Table 1**). The variants, as well as SNPs in non-coding regions, were almost entirely located on a 292 kb region of contig DS_499598, which contained 20 of the variant genes (**Figure 3**) with the position, gene ID, and SNP variation in genes listed in **Supplementary Table S1**. Further bioinformatic analysis identified three clustered genes within this 292 kb region which exhibited the highest variant ratios (all values > 0.9) i.e. the highest homozygosity for the SNP reads (**Figure 5A**: **Supplementary Table S1**),

### Use of BSA and NGS With 80 Sexual Progeny per DNA Pool

**Figure 4:**
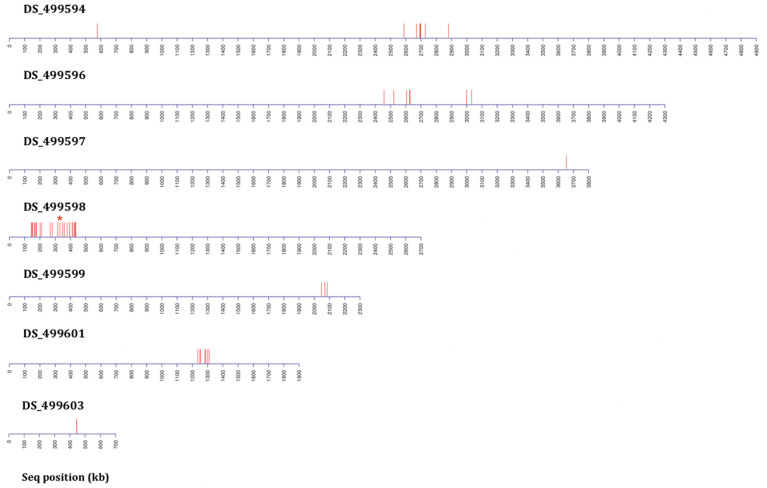
Distribution plots of SNP variants identified from bioinformatic analysis mapped onto *A. fumigatus* JGI reference A1163 genome contigs (not to scale) from bulk segregant analysis (BSA) applied following two rounds of backcrossing (BC2) using 80 progeny per BSA pool. Each red bar represents one or more (if in close proximity) variant sites exhibiting a consistent difference between the sensitive and resistant groupings. Data includes sites present in gene coding and non-coding regions. The red asterisk above contig DS_499598 indicates the position of the M220K causal variant in *cyp51A*.

**Figure 5:**
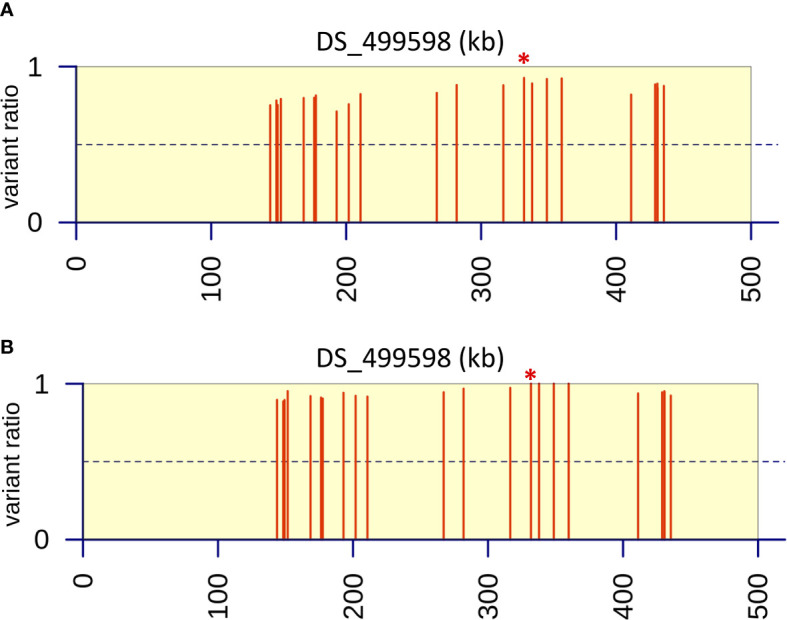
Variant ratio plots to show extent of heterozygosity or homozygosity at SNPs identified on contig DS_499598 from bioinformatic analysis. **(A)** Data analysis of resistant parent and progeny pool (n=40) derived after six back crosses (BC6). **(B)** Data analysis of resistant parent and progeny pool (n=80) derived after two back crosses (BC2). Values were calculated as the ratio of the average coverage supporting that variant compared to the average total coverage at that given position across the three replicates in the progeny pools. The red asterisk indicates the position of the M220K causal variant in *cyp51A*.

In order to test whether using more progeny per DNA pool could significantly increase the efficiency of BSA in identification of candidate regions, an additional round of BSA and bioinformatic analysis was undertaken using progeny from the 2^nd^ backcross (BC2) but this time pooling 80 resistant and 80 sensitive progeny. Bioinformatic analysis of genomic data from the resistant progeny in comparison to the sensitive parent, sensitive progeny and the reference A1163 genome, revealed 29 genes demonstrating SNPs between the different sensitive and resistant groupings (**Table 1**). The majority (83%) of the variants were located on a 292 kb region of contig DS_499598, which contained the same 20 variant genes as seen from BC6, although SNP variants (including those not in coding regions) were located across 7 contigs (**Figure 4**) with the position, gene ID, and SNP variation in genes listed in **Supplementary Table S2**. Further bioinformatic analysis identified four clustered genes within this 292 kb region which all exhibited variant ratios of 1.0 i.e. complete homozygosity for the SNP reads in the resistant parent and pool (**Figure 5B**: **Supplementary Table S2**),

### Identification of Genetic Basis of Azole Resistance

The BSA and associated bioinformatic analysis of progeny from the 6^th^ backcross (BC6; using 40 progeny per pool) and the 2^nd^ backcross (BC2; using 80 progeny per pool) both identified a 292 kb region of the genome on contig DS_499598 containing 20 linked genes which showed consistent SNP variants between the resistant progeny compared to the sensitive progeny, sensitive parent and sensitive A1163 genome reference strain. This list of candidate genes with a role in resistance to itraconazole included genes with known functions based on BLAST homology and also a series of hypothetical genes of unknown function (**Supplementary Tables S1** and **S2**). A clustered subset of three and four genes from BC6 and BC2, respectively, exhibited the highest homozygosity within the resistant parent and progeny groups. Close inspection of these particular candidate genes revealed that they included the *cyp51A* gene (AFUB_063960), which was present in the central third of the region (ca. 188 kb into the 292 kb region), and which contained a T to A variant that would result in an amino acid change of methionine to lysine (M220K) in the translated CYP51A protein. An M220K substitution in *cyp51a* has already been described by Mellado et al. (2004) who reported that substitutions of methionine to lysine, threonine or valine at position 220 of *cyp51A* greatly reduced susceptibility to all triazole antifungals. These substitutions resulted in *A. fumigatus* isolates with resistance to itraconazole with MICs >8 mg/L (Mellado et al., 2004). Due to the monogenic pattern of segregation of the resistance phenotype it was therefore concluded that this M220K mutation explained the genetic basis of azole resistance in parental isolate 47-308 and the resistant progeny from the various rounds of back crossing, with no further experimental validation required.

## Discussion

The discovery that *Aspergillus fumigatus* has a functional, heterothallic sexual cycle, combined with the observation that sexual fertility is widespread in global populations (Swilaiman et al., 2020; Korfanty et al., 2021), has important implications for the possibility of gene flow within populations regarding detrimental traits such as resistance to antifungal drugs and virulence. However, the presence of a sexual cycle also offers a valuable tool for genetic analysis. In the present study we evaluated the possible application of bulk segregant analysis (BSA) combined with next-generation sequencing of the progeny pools for genetic analysis of monogenetic traits in *A. fumigatus*, using the trait of resistance to the azole antifungal drug itraconazole as a model. This revealed that BSA could be used to identify a region within the genome of below 300 kb in size which contained candidate genes of interest, with both the use of increasing numbers of backcrosses and increased numbers of progeny (within the respective resistant and sensitive pools) increasing the efficiency of the BSA process as will now be described.

Bulk segregant analysis was first proposed and used to study resistance to fungal downy mildew disease in the lettuce *Lactuca sativa* (Michelmore et al., 1991). The original methodology relied on the screening of progeny pools using RAPD fingerprint markers in order to detect a marker linked to the trait of interest. It was possible to reliably identify a 25-centimorgan region containing the resistance allele, although the precise allele involved was not identified and further work involving rounds of chromosome walking was recommended. Other DNA fingerprint markers such as AFLP and RAD were also subsequently used (Jurgenson et al., 2002; Baird et al., 2008; Dettman et al., 2010) and even hybridization against tiling arrays (Reisser et al., 2013). More recently, BSA has been updated to incorporate next-generation sequencing (NGS) of the progeny pools and has been applied in various ways in the field of fungal genetics. Wenger et al. (2010) used BSA and NGS to identify candidate genes involved with xylose utilization in *Saccharomyces cerevisiae*. Pomraning et al. (2011) used BSA and high-throughput sequencing to identify a temperature sensitive mutant through crossing of related strains of *Neurospora crassa*, as did Nowrousian et al. (2012) when employing BSA to identify developmental and spore color genes in *Sordaria macrospora.* By contrast, Niu et al. (2016) used a progeny pool from parasexual crossing of very closely related strains of *A. niger* to identify a mutation responsible for a non-acidifying phenotype by direct comparison to a parental strain. Most recently Heller et al. (2016; 2018) and Goncalves et al. (2019) applied BSA and NGS to investigate the genetic basis of allorecognition processes in *N. crassa*, and were able to identify genomic regions of interest with candidate genes. In a parallel approach to BSA, Bok et al. (2014) used successive backrosses and then sequence comparison of just two progeny and the parental line to identify an oxidative stress transcription factor in *A. nidulans*. In all applications of BSA it is self evident that great care must be taken to avoid sample contamination, as misleading data could arise if a sample with a particular phenotype was accidentally included in the wrong pool. This is particularly a potential risk with larger pool numbers.

In the present study we used pooled offspring from conventional sexual crosses to conduct BSA. Progeny were distinguished by resistance or sensitivity to itraconazole, which was shown to be a monogenic Mendelian trait based on progeny segregation patterns. A dedicated bioinformatic pipeline was designed especially for this study to allow first the identification of SNPs of interest (whether in coding or non-coding regions) which were consistently present in the pool of resistant progeny and the resistant parent but absent from the pool of sensitive progeny and the sensitive parent (**Supplementary Figure 1**). The pipeline then focussed on the subset of SNPs present within coding regions as a starting point to try to identify the possible causal basis of resistance. In addition an extra rule was applied that any SNP should also be absent from the reference A1163 and Af293 genomes used for alignment because these were both itraconazole sensitive. This new pipeline was considered to have advantages over previously described methods because it introduced extra rules for required differences between the progeny pools and the parental strains. This helped allow the use of parental lines which were potentially highly divergent, unlike many of the other previous BSA studies with filamentous fungi which used often highly related strains in the original crosses (e.g. Pomraning et al., 2011; Nowrousian et al., 2012; Niu et al., 2016). The use of variant analysis to identify SNPs with high homozygosity values in the parent and pool of interest was also facilitated by the ready public access of standard bioinformatic tools to allow such analyses. In preliminary analysis a check was also made for the possible presence of deleted regions of the genome specifically in the resistant pools and parent that might be correlated with resistance, but no such region(s) was found.

BSA and associated bioinformatic analysis was first undertaken with pools of 40 resistant and 40 sensitive F1 progeny direct from the initial cross between the itraconazole resistant (47-308) and sensitive (47-51) parents. It was hoped that this might allow sufficient resolution of a genomic region of interest that contained an SNP(s) responsible for the resistance phenotype. However, SNPs were detected over 8-11 contigs/chromosomes and between 123-150 candidate genes were identified (based on alignment to JGI and NCBI genome reference databases, respectively) i.e. there was no clear single region or gene of interest. Therefore, a series of rounds of backcrosses were undertaken between resistant offspring and the sensitive parent in order to increase the isogenic nature of the offspring, which would then be expected to increasingly differ only in a region linked to the trait of interest, in this case azole resistance. Thus, backcrossing was used to drive the segregation of chromosomes without causal variation, as they will become parental, combined with backcrossing serving to increase the opportunity for cross over and recombination to reduce the size of the genomic region containing the trait of interest. The subsequent BSA of progeny pools after successive backcrosses did indeed show increased ability to identify a region and candidate genes of interest when using 40 progeny per resistant and sensitive pool. BSA and bioinformatic analysis using progeny from the third backcross identified a lower number of 46-60 genes which showed SNPs between the resistant and sensitive pools and parents (alignment to JGI and NCBI databases, respectively), with a single region of the genome containing 41 of the variant genes, although there were still consistent SNPs present on some other regions of the genome (Ashton, 2018). By contrast, BSA and bioinformatic analysis using progeny from the sixth backcross clearly distinguished a single 292 kb region of the genome containing a lower number still of 20 genes which showed SNPs between the resistant and sensitive pools and parents. A small number of other SNPs were also present in the genome but there was no clear linkage with neighbouring genes, as would be expected from BSA. A final round of BSA and bioinformatic analysis was then undertaken but this time using an increased number of 80 progeny per segregant pool from the second backcross. The second backcross was chosen as a balance between time demands of further backcrossing and time required to isolate increased numbers of single ascospore progeny in *A. fumigatus* (Ashton and Dyer, 2019). This proved very effective, with 29 genes demonstrating SNPs between the different sensitive and resistant groupings detected using progeny from just the second backcross. Significantly, the majority of variant SNPs were again located on the identical 292 kb region of the genome as found after six rounds of backcrossing, which contained the same 20 variant genes.

Subsequent bioinformatic analysis of the 292kb region identified the presence of four clustered genes which showed the highest variant ratios (i.e. homozygosity) within the region. These included the *cyp51A* gene, with the presence of a T to A variant SNP in *cyp51A* within the resistant progeny and parent as compared to the sensitive pools and parent. This led to a methionine to lysine (M220K) change in the CYP51A protein. Given that an identical M220K substitution in *cyp51a* has already been reported and experimentally verified by Mellado et al. (2004) as leading to greatly reduced susceptibility to triazole antifungals in *A. fumigatus*, it was clear that this mutation was mainly, if not solely, responsible for the itraconazole resistance seen in isolate 47-308. Therefore, no further experimental validation of the candidate genes was required.

The failure to detect a novel genetic form of resistance to itraconazole was perhaps disappointing and the fact that this SNP had not been identified during preliminary screening indicates a possible mixed source isolate. However, taken as a whole, results presented in the present study clearly illustrate that BSA in conjunction with the dedicated bioinformatic pipeline can be used as a very valuable tool in *A. fumigatus* to investigate the genetic basis of a trait showing monogenic inheritance but for which the causal gene/SNP is unknown. The results of the present study are also of great value in demonstrating that the efficiency of BSA can be improved both by employing successive rounds of backcrossing and also by increasing the number of progeny in the BSA pools. The latter is consistent with mathematical modelling of the predicted size of genomic blocks obtained versus number of progeny screened as hypothesized by Pomraning et al. (2011) and Ashton (2018), who both described a steep decrease in the size of the identified linkage region of interest as the progeny pool size increased to 40, but a less pronounced effect as pool size increased up to 100 or more. It is noted that other recent fungal BSA and NGS studies have used pool sizes between 40-78 (Pomraning et al., 2011; Nowrousian et al., 2012; Niu et al., 2016; Heller et al., 2018; Goncalves et al., 2019). During the course of the present study Losada et al. (2015) also suggested that BSA could be a useful new technique in *A. fumigatus* as part of a study on construction of isogenic mating strains. However, in a preliminary application many SNPs did not follow expected distribution patterns and it was cautioned that certain causal genes might be overlooked.

It was very interesting to note that despite the use of six rounds of backcrossing, or the use of a high number (80) progeny per pool after a second backcross (BC2), that it was not possible to identify a single unique SNP or gene of interest by BSA. Instead the same 292 kb region containing 20 candidate genes, with a clustered subset of three-four genes with highest variant homozygosity, was identified by both approaches, with the only difference being that the BC2 pools included two additional small linked regions other than the 292 kb region; these extra regions were presumably broken up by cross over and recombination events between the second and sixth backcross. Indeed, this same linked region on contig DS_499598 was apparent even in the BSA of the F1 progeny, albeit with many additional candidate regions present elsewhere in the genome (Ashton, 2018). Thus, the present results indicate that there can be regions within the *A. fumigatus* genome which undergo relatively little crossover, leading to tightly linked regions as seen on contig DS_499598, with consistent segregation and preservation of this region during sexual recombination between isolates. However, this does not necessarily imply a low recombination rate in the genome of *A. fumigatus* as a whole. Indeed, the fact that most genes in the 292 kb region did not show total homozygosity within the resistant pool is consistent with very occasional cross over in this region in a subset of the progeny used to construct the pools. It was telling to note that only four genes from the BC2 analysis (including *cyp51A*) showed complete homozygosity for the SNP reads in the resistant parent and progeny pool. Furthermore, it has very recently been reported that one cross of *A. fumigatus* exhibited an exceptionally high rate of meiotic recombination with ca. 29 crossovers per chromosome, representing possibly the highest known crossover rate of any eukaryotic species (Auxier et al., 2022). Intriguingly, the authors also reported very rare, but detectable, evidence of recombination within the region of the *cyp51A* gene, possibly explained by different extents of divergence between the crossing parents used in our respective studies. Also highly relevant to the present observations are the findings of Gell et al. (2020) who recently reported that recombination and cross over during sexual reproduction of the related species *A. flavus* was not random across the genome but instead there were both hotspots and coldspots, and that there were an estimated 15-20 recombination events across the genome in a typical mating event.

Therefore, these results indicate that the BSA approach here described for *A. fumigatus* will not be able to identify a single unique SNP or gene of interest unless a much increased number of progeny and/or backcrosses is used. Consistent with our results, Losada et al. (2015) found that SNP differences were still present in progeny of *A. fumigatus* throughout the genome even after nine successive backcrosses, although differences were mainly limited to a small number of blocks. Nevertheless, the size of the genomic region that might be identified through BSA (e.g. 300 kb based on the present data) is argued to be small enough to identify a manageable number of candidate genes, particularly if a subset of genes with highest homozygosity can be focused on. However, it is cautioned that larger linked blocks might be encountered elsewhere in the genome, although there may also be more beneficial smaller linked blocks as indicated by the study of Auxier et al. (2022). In parallel studies with *N. crassa* using conventional sexual crosses Heller et al. (2016) were able to identify a ca. 100 kb region by BSA, Heller et al. (2018) identified a 180 kb region, whilst Goncalves et al. (2019) were able to identify a 1Mb region by BSA (using pools of 46-50 progeny from a cross or single backcross). Furthermore, although the bioinformatic pipeline used in the present study focussed on SNPs within coding regions, the identification of such a genomic region would allow thorough sequence analysis which would identify features such as the TR34 and TR46 tandem repeats in the promoter regions of *cyp51A*, which are an important causal reason of azole resistance in certain isolates of *A. fumigatus* (Mellado et al., 2007; Snelders et al., 2015). Once candidate genes or such indels have been identified, further genetic manipulation can then be carried out (e.g. *via* conventional GM or CRISPR/Cas9 methodology) to pinpoint the gene involved and fully characterise the mechanism of resistance.

Looking ahead to the future use of BSA for analysis of monogenic traits in *A. fumigatus* and fungal genetics more generally, the balance of whether to use an increased number of progeny in pools and/or to perform successive rounds of backcrossing may be influenced in part by time-considerations. In situations where a shorter time frame is required, using a larger number of progeny but fewer backcrosses might yield similar results as repeated backcrossing. Meanwhile, the bioinformatics pipeline developed for the current study may be widely applicable for BSA of conventional sexual crosses in other fungal species where a monogenic trait is being investigated. Finally, in the specific case of *A. fumigatus* other methods of genetic analysis such as those involving quantitative trait loci (QTL; Christians et al., 2011) are now possible following the ability to perform sexual crosses *in vitro*. Conventional QTL analysis will provide a valuable complement to BSA in situations where a trait, such as resistance to antifungals, is found to be polygenic rather than monogenic in nature. Indeed, newer methods termed extreme QTL mapping (X-QTL) (Ehrenreich et al., 2010) and QTL-seq (Takagi et al., 2013) have been developed for identification of polygenic traits, involving a hybrid BSA approach in which pools are selected from the extreme ends of a segregating population and a sliding window analysis can then be used to identify divergent loci and allelic frequency differences between the bulks (Ehrenreich et al., 2010; Magwene et al., 2011; Swinnen et al., 2012; Takagi et al., 2013). Both BSA and QTL analysis can complement methods such as *in vitro* experimental evolution and genome-wide association studies (GWAS), which have also recently been utilised to investigate virulence and disease progression (Kowalski et al., 2019) and the basis of azole resistance in *A. fumigatus* (Fan et al., 2021).

## Data Availability Statement

The datasets presented in this study can be found in online repositories. The names of the repository/repositories and accession number(s) can be found below: NCBI, GSE193956.

## Author Contributions

PSD, GDA, FS, and MB designed the study. GDA, NH and SM performed the experiments. MB, FS, and VW were involved with the bioinformatic analysis. PSD and GDA wrote the manuscript. SMTC, WJGM, PEV, and PSD contributed materials. VW and FS contributed to editing of the article. All authors contributed to the article and approved the submitted version.

## Funding

This work was supported by grants from the Natural Environment Research Council UK (NE/P000916/1), the Wellcome Trust (219551/Z/19/Z), and the Biotechnology and Biological Sciences Research Council (BB/M008770/1) through a DTP PhD studentship.

## Conflict of Interest

The authors declare that the research was conducted in the absence of any commercial or financial relationships that could be construed as a potential conflict of interest.

## Publisher’s Note

All claims expressed in this article are solely those of the authors and do not necessarily represent those of their affiliated organizations, or those of the publisher, the editors and the reviewers. Any product that may be evaluated in this article, or claim that may be made by its manufacturer, is not guaranteed or endorsed by the publisher.
