# Supplementary material for: Use of Bulk Segregant Analysis for Determining the Genetic Basis of Azole Resistance in the Opportunistic Pathogen Aspergillus fumigatus
Source: Front Cell Infect Microbiol. 2022 Apr 5;12:841138. doi: 10.3389/fcimb.2022.841138 (PMC9069965; doi:10.3389/fcimb.2022.841138)
Supplement: Supplementary file 1 [file Image_1.pdf]

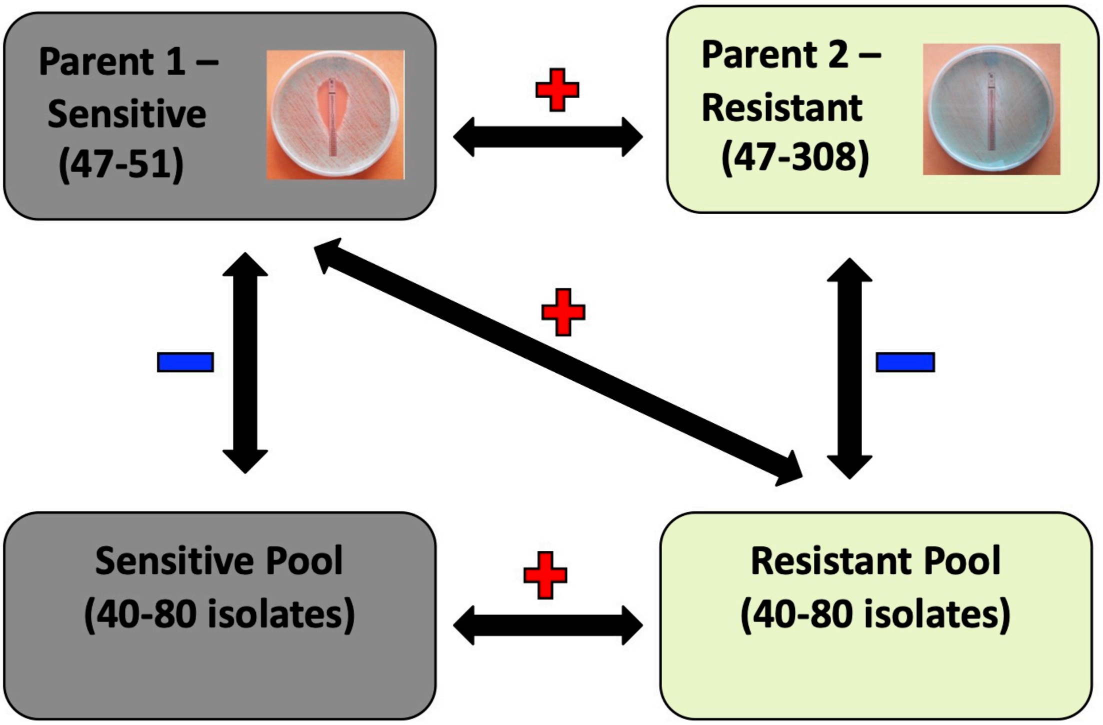


**SUPPLEMENTARY FIGURE S1** - Representation of the rules used for bulk segregant analysis. Here (+) indicates differences in genome sequence that will be consistently present, whilst (-) indicates similarities to be ignored. Thus the analysis will identify sequencing variant(s) between the resistant and sensitive pools, which are also evident between the resistant pool and sensitive parent, whilst ignoring similarities between the resistant and sensitive pools and their respective parental genomes.
