# Supplementary material for: Use of Bulk Segregant Analysis for Determining the Genetic Basis of Azole Resistance in the Opportunistic Pathogen Aspergillus fumigatus
Source: Front Cell Infect Microbiol. 2022 Apr 5;12:841138. doi: 10.3389/fcimb.2022.841138 (PMC9069965; doi:10.3389/fcimb.2022.841138)
Supplement: Supplementary file 2 [file Image_2.pdf]

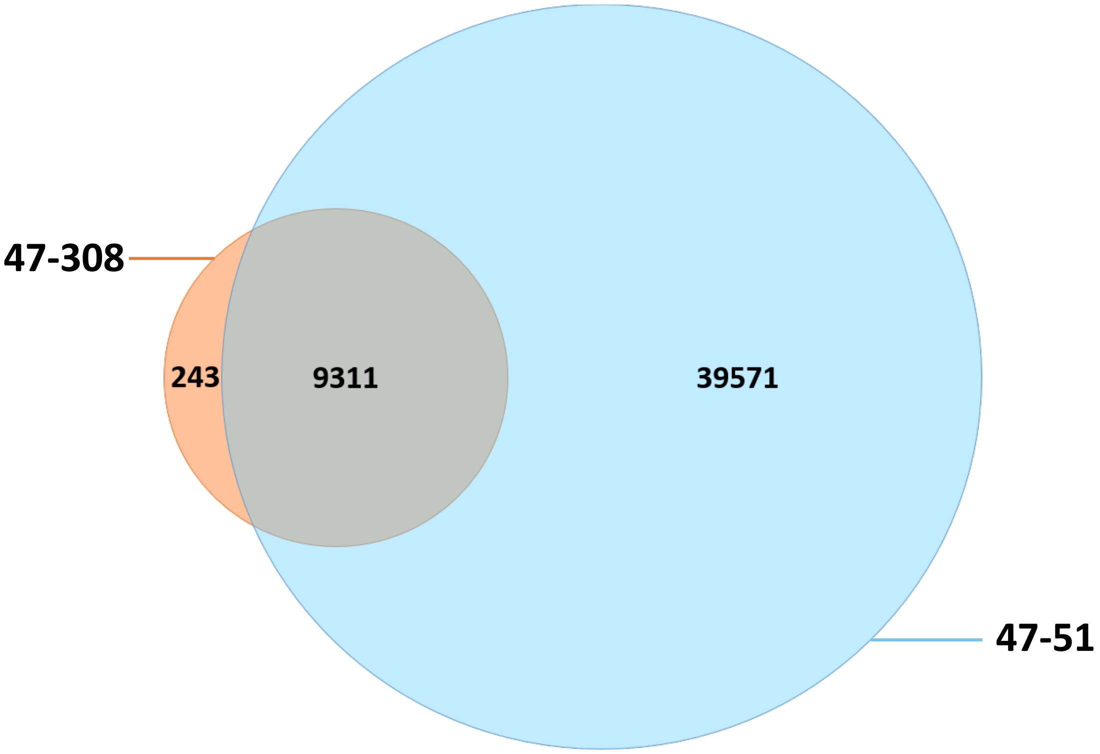

SUPPLEMENTARY FIGURE S2 - Representation of SNP variants present between *Aspergillus fumigatus* parental isolates 47-51 (right hand side) and 47-308 (left hand side) when compared to the A1163 reference genome. 47-51 shows a total of 48,882 SNP variants and 47-308 exhibits 9,554 variants from the A1163 reference genome (https://mycocosm.jgi.doe.gov/Aspfu_A1163_1/Aspfu_A1163_1.home.html), respectively, with 9,311 shared SNPs compared to the A1163 reference genome.
