# Supplementary material for: Use of Bulk Segregant Analysis for Determining the Genetic Basis of Azole Resistance in the Opportunistic Pathogen Aspergillus fumigatus
Source: Front Cell Infect Microbiol. 2022 Apr 5;12:841138. doi: 10.3389/fcimb.2022.841138 (PMC9069965; doi:10.3389/fcimb.2022.841138)
Supplement: Supplementary file 3 [file Image_3.pdf]

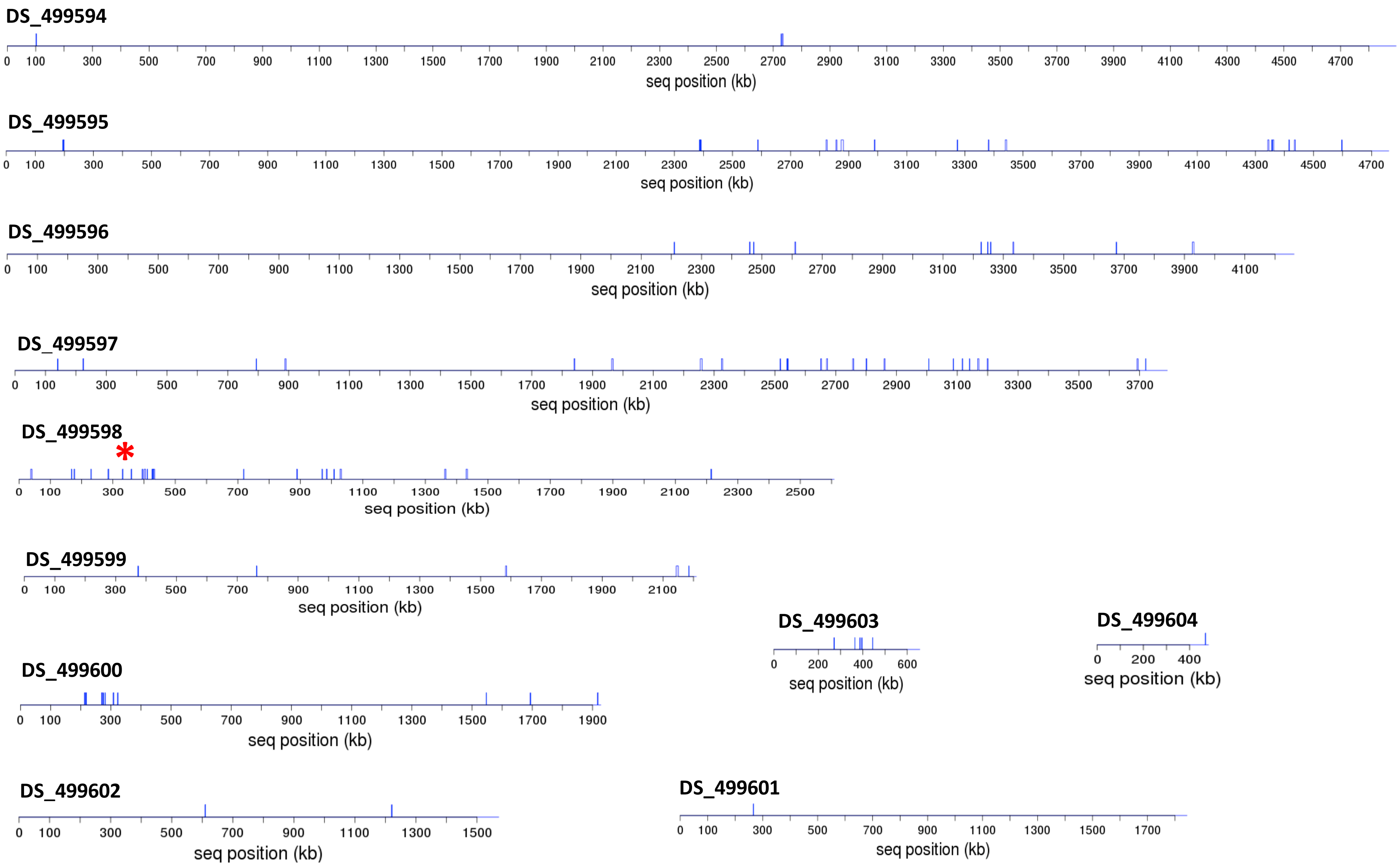

SUPPLEMENTARY FIGURE S3 - Distribution plots of identified SNP variants mapped onto the *A. fumigatus* JGI reference A1163 genome contigs (not to scale) during the first round (F1) of BSA sequencing using 40 progeny per BSA pool. Each blue line identifies at least one SNP in a 10 kb region of a gene or non-coding region exhibiting a consistent difference between the sensitive and resistant groupings (not all SNPs are shown). Note that a slightly different bioinformatic approach was used in later BC2, BC4 and BC6 analyses, meaning that certain differences in SNP detection are evident. The red asterisk above contig DS_499598 indicates the position of the M220K causal variant in *cyp51A*.
