# Supplementary material for: Use of Bulk Segregant Analysis for Determining the Genetic Basis of Azole Resistance in the Opportunistic Pathogen Aspergillus fumigatus
Source: Front Cell Infect Microbiol. 2022 Apr 5;12:841138. doi: 10.3389/fcimb.2022.841138 (PMC9069965; doi:10.3389/fcimb.2022.841138)
Supplement: Supplementary file 4 [file Image_4.pdf]

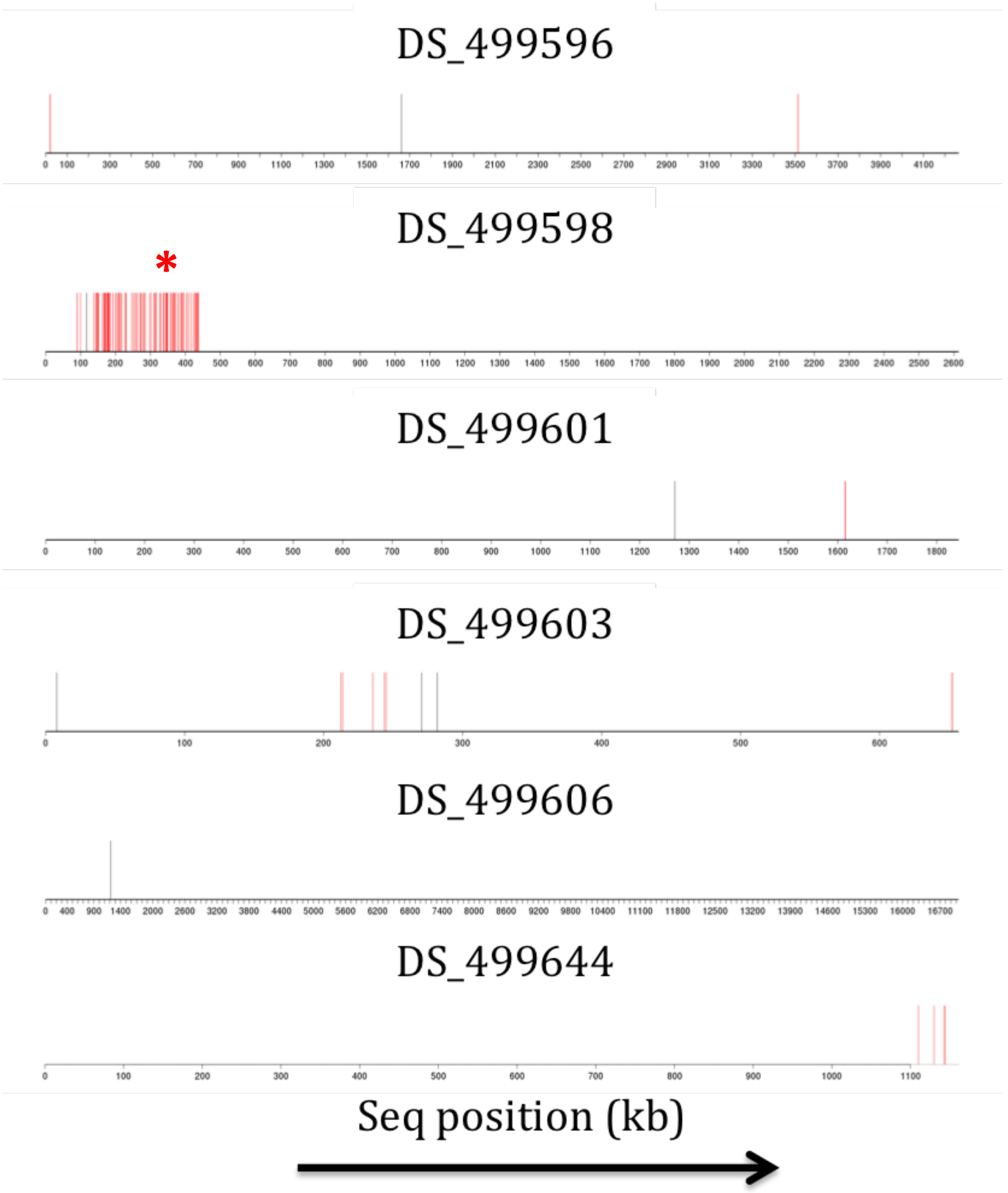

SUPPLEMENTARY FIGURE S4 - Distribution plots of identified SNP variants mapped onto the *A. fumigatus* JGI reference A1163 genome contigs (not to scale) from the third round of backcrossing (BC3) of BSA sequencing using 40 progeny per BSA pool. Each bar represents one or more (if in close proximity) variant sites exhibiting a consistent difference between the sensitive and resistant groupings. Data includes sites present in gene coding and non-coding regions. The red asterix above contig DS_499598 indicates the position of the M220K causal variant in *cyp51A*.
