# Supplementary material for: Use of Bulk Segregant Analysis for Determining the Genetic Basis of Azole Resistance in the Opportunistic Pathogen Aspergillus fumigatus
Source: Front Cell Infect Microbiol. 2022 Apr 5;12:841138. doi: 10.3389/fcimb.2022.841138 (PMC9069965; doi:10.3389/fcimb.2022.841138)
Supplement: Supplementary file 5 [file Table_1.docx]

**Supplementary Table S1** Details of 22 genes identified in the resistant progeny from the 6^th^ backcross (BC6) with sensitive parent 47-51, which show consistent SNP variation when compared to the sensitive progeny and parent isolate and reference genome A1163 (using 40 progeny per pool). The contig, position, gene ID, gene name, reference and SNP change are listed. Where a gene name was not available, the putative gene function is listed.

| **Contig** | **Position** | **Gene ID** | **Gene** | **Ref/**  **Change** | **Variant**  **Ratio^1^** |
| --- | --- | --- | --- | --- | --- |
| DS_499596 | 2628539 | AFUB_043230 | Hypothetical | A/G | 0.58 |
| DS_499598 | 143724 | AFUB_063310 | Fungal specific transcription factor | G/A | 0.75 |
| DS_499598 | 148327 | AFUB_063320 | Flavin-binding mono oxygenase | A/G | 0.78 |
| DS_499598 | 149261 | AFUB_063330 | Hypothetical | C/T | 0.75 |
| DS_499598 | 151616 | AFUB_063350 | *agp2* | A/G | 0.79 |
| DS_499598 | 168480 | AFUB_063420 | Hypothetical | T/C | 0.80 |
| DS_499598 | 176285 | AFUB_063470 | *gar2* | T/C | 0.79 |
| DS_499598 | 177560 | AFUB_063470 | *gar2* | C/G | 0.81 |
| DS_499598 | 192943 | AFUB_063540 | Transcription binding | T/C | 0.71 |
| DS_499598 | 201933 | AFUB_063560 | dDENN domain protein | G/C | 0.76 |
| DS_499598 | 210643 | AFUB_063570 | *mlh3* | C/G | 0.82 |
| DS_499598 | 267123 | AFUB_063720 | Acid sphingo-myelinase | G/A | 0.83 |
| DS_499598 | 281565 | AFUB_063770 | GPI anchored cell wall protein | C/A | 0.88 |
| DS_499598 | 316444 | AFUB_063920 | Coenzyme A synthetase | T/C | 0.88 |
| DS_499598 | 331794 | AFUB_063960 | *cyp51A* | T/A | 0.93 |
| DS_499598 | 337780 | AFUB_063980 | Hypothetical | C/A | 0.89 |
| DS_499598 | 348715 | AFUB_064030 | Hypothetical | A/T | 0.91 |
| DS_499598 | 359699 | AFUB_064070 | Hypothetical | T/A | 0.92 |
| DS_499598 | 411115 | AFUB_064260 | *sts1* | T/G | 0.82 |
| DS_499598 | 428931 | AFUB_064340 | Ribsomal protein S25 | A/G | 0.89 |
| DS_499598 | 430556 | AFUB_064350 | *sog2* | T/G | 0.89 |
| DS_499598 | 430665 | AFUB_064350 | *sog2* | C/G | 0.89 |
| DS_499598 | 435376 | AFUB_064360 | Serine carboxy-peptidase | A/T | 0.87 |
| DS_499603 | 444821 | AFUB_099850 | Hypothetical | G/A | 0.60 |

^1^Variant ratio provided an indication of the extent of homozygosity or heterozygosity at a particular SNP. Values were calculated as the ratio of the average coverage supporting that variant compared to the average total coverage at that given position across the three replicates in the progeny pools.
